# Structure-Aware Noise Scheduling for Fixed-View Visual Sensor Reconstruction

**DOI:** 10.3390/s26165304

**Published:** 2026-08-21

**Authors:** Xingyu Lu, Zengshan Yao

**Affiliations:** Faculty of Information Science and Engineering, Ocean University of China, Laoshan Campus, Beihaiyuan, No. 238 Songling Road, Shazikou Subdistrict, Laoshan District, Qingdao 266100, China; 18524440390@163.com

**Keywords:** visual sensors, fixed-view imaging, diffusion reconstruction, residual analysis, noise scheduling, MVTec AD, structure preservation

## Abstract

Fixed-view visual sensors require normal-image reconstruction that preserves structural detail while exposing a local deviation in a residual map. Standard denoising diffusion probabilistic models (DDPMs) use spatially uniform corruption. We study an image-derived, structure-aware noise schedule for reference-image reconstruction, where the input image is available and the same importance map can be fixed in the forward and reverse processes. A matched three-seed diagnostic further isolates the effect of the semantic-training/gradient-reverse map substitution and of a lower bound on local noise. Map consistency recovers much of the unconditional FID loss, but neither it nor the attenuation floor yields a universal advantage over DDPM. The six-category MVTec AD residual study is likewise category dependent: the gradient/edge special case improves selected texture-localization outcomes but degrades several object-level outcomes. We therefore present the method as a reconstruction diagnostic, not as a competitive industrial anomaly detector or a universally superior generator.

## 1. Introduction

Fixed-view visual sensors are routinely used to compare a current image with the normal appearance of a product, material, or component. A reconstruction model provides one possible reference: a local deviation can be exposed by the residual between the observed and reconstructed image. In this setting, the input image is known at inference time. The central question is therefore not unrestricted image generation, but how the corruption-and-reconstruction schedule changes the retention of normal structure and the localization of a residual.

Denoising diffusion probabilistic models (DDPMs) [1] use a spatially uniform Gaussian schedule. This is appropriate when all locations should be treated symmetrically, but it does not distinguish a product boundary, a repetitive material pattern, and a smooth normal interior. Edge-preserving diffusion [2] modifies the noise scale using an image gradient, inspired by the Perona–Malik diffusivity [3]. The idea is attractive for visual reconstruction: reducing intermediate corruption at a strong normal structure could yield a different reconstruction residual around a local deviation without changing the U-Net architecture.

We study a general image-derived structure-importance map, which can combine a gradient with an optional semantic foreground cue. The central empirical setting is deliberately narrower than the general formula. For fixed-reference reconstruction, the observed image is available when the map is constructed; the same map can be held fixed during noising and reverse reconstruction. This avoids the train–test substitution required by unconditional sampling, where a semantic training map would otherwise be replaced by a gradient of an estimated clean image. The completed MVTec AD study evaluates the gradient/edge-preserving special case only ($\alpha_{1}=1$, $\alpha_{2}=0$). It therefore tests a structure-aware residual mechanism, not the semantic foreground term as an industrial inspection method.

The evaluation combines a focused schedule diagnostic with the sensor-facing residual study. A matched three-seed AFHQ ablation separates the SAM2-training/gradient-reverse substitution from the spatial schedule itself, and an inference-time floor sweep measures how bounded attenuation changes FID and ECS. The six-category MVTec AD evaluation uses normal-only category-specific training and compares three textures with three object classes. DDPM and the gradient schedule are matched in architecture, resolution, optimizer, reconstruction strength, and random seeds. The results are deliberately mixed: map consistency restores much of the unconditional fidelity loss without surpassing DDPM FID, and selected texture-localization outcomes improve while several object categories degrade. These negative outcomes delimit the contribution rather than being exceptions to conceal.

Contributions.

The article makes the following bounded contributions.

1. **Fixed-reference formulation.** We formulate a structure-aware diffusion reconstruction schedule in which an importance map derived from the observed reference image is available on both sides of the reconstruction process.
2. **Map-source and attenuation diagnostics.** A matched three-seed ablation tests the proxy-consistent alternative to SAM2-map training/gradient-map sampling; an inference-time noise-floor sweep tests attenuation strength. Both identify sources of FID loss without establishing an unconditional advantage over DDPM.
3. **Fixed-reference mechanism and residual study.** A controlled reference-editing check uses the known map in both directions. We also provide three-seed, normal-only DDPM and gradient-schedule results on six MVTec AD categories, including five residual metrics, strength sensitivity, and computational cost.
4. **Evidence boundary.** We interpret the study as a fixed-view reconstruction diagnostic. It does not claim a universal unconditional-generation advantage, a semantic-map MVTec result, or replacement of a mature industrial anomaly detector.

Section 2 positions the work among diffusion reconstruction, object-aware reconstruction, and residual inspection. Section 3 defines the schedule and fixed-reference protocol. Section 4 reports the schedule diagnostics, controlled reference-edit check, and matched MVTec experiments. Section 5 discusses scope, limitations, and practical interpretation.

## 2. Related Work

### 2.1. Diffusion Reconstruction and Spatial Schedules

DDPMs [1] learn to invert a prescribed Gaussian corruption process. In an image-to-image setting, SDEdit [4] starts a reverse trajectory from a noised reference image and thereby trades fidelity to that reference against editing freedom. This reference availability is important here: it permits a map computed from the observed image to be used consistently throughout the reconstruction. By contrast, unconditional sampling has no such reference and requires a reverse-time proxy for a clean-image-derived map.

Spatially adaptive diffusion schedules have been proposed to preserve strong visual structure. Edge-preserving diffusion [2] uses a Perona–Malik-inspired scale driven by an image gradient, attenuating noise at high-gradient locations. Other work introduces structure through blur-based forward processes [5,6] or through reference-conditioned reverse models [7,8]. Our setting does not add a conditioning network. It examines whether an image-derived scale changes a fixed-reference diffusion reconstruction and its residual.

### 2.2. Object-Aware Reconstruction Priors

Object-aware priors can make a reconstruction process allocate attention or uncertainty non-uniformly. Li et al. [9] introduced OUGS, which estimates object-aware uncertainty in 3D Gaussian Splatting and uses it to select informative views for scene reconstruction. The present work is distinct in both representation and objective: OUGS selects views for 3D reconstruction, whereas we apply a 2D pixelwise schedule to a known fixed-view image. Nevertheless, OUGS motivates the broader premise that object or structure awareness should be made explicit when a reconstruction pipeline decides where uncertainty is allowed to accumulate. Our optional foreground term is a general formulation of that premise; the completed MVTec experiment below intentionally restricts itself to the gradient term.

Semantic segmentation has also been used as a spatial prior in generative modeling, for example through SPADE [10], semantic editing [11], and ControlNet-style conditioning [8]. These approaches typically introduce an explicit semantic condition in the generator. Here, a foreground map only controls the noise scale. A semantic-map result must therefore demonstrate that the map remains available and consistent during reconstruction. Section 4 separates a proxy-consistency diagnostic for unconditional sampling from the fixed-reference reconstruction setting in which the observed-image map remains available.

### 2.3. Residual-Based Visual Inspection

MVTec AD [12] is a standard normal-only visual-inspection benchmark with image-level labels and pixel annotations reserved for test evaluation. Reconstruction residuals are one way to expose a deviation, but high-performing industrial systems commonly optimize discriminative feature-space anomaly scores. PaDiM [13] models normal patch-feature distributions, and PatchCore [14] uses a memory of normal features. These approaches differ in objective and reporting protocol from the residual reconstruction used here. We consequently use MVTec to stress-test the effect of a schedule on residual maps, not to claim a head-to-head detector comparison.

## 3. Fixed-Reference Structure-Aware Reconstruction

**Figure 1:**
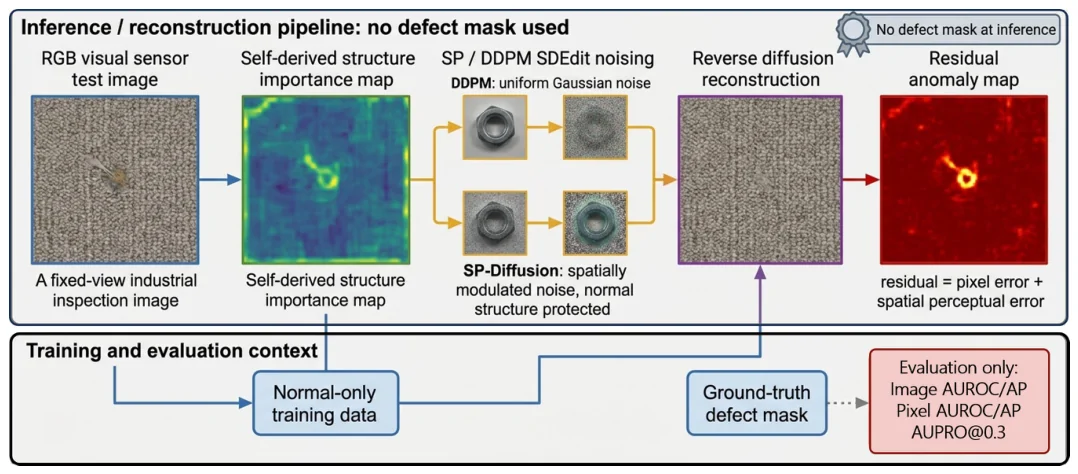
Fixed-reference reconstruction protocol. An image-derived structure map is formed from the observed visual-sensor image, retained for the reconstruction trajectory, and used to produce a residual map. The defect mask is unavailable to the reconstruction and is used only for test evaluation. In the completed MVTec study, the structure map is gradient-based ($\alpha_{1}=1$, $\alpha_{2}=0$).

Figure 1 outlines the fixed-reference reconstruction protocol.

### 3.1. Importance Map and Special Cases

Let $x_{0}\in R^{C\times H\times W}$ be a reference image observed by a fixed-view sensor. We define the general structure-importance map

(1) $$I_{\mathrm{ref}}=\mathrm{Norm}\;\left(\alpha_{1}{∥\nabla x_{0}∥}_{\mathrm{norm}}+\alpha_{2}M\left(x_{0}\right)\right),\;I_{\mathrm{ref}}\in {\left[0,1\right]}^{C\times H\times W},$$

where $∥\nabla x_{0}{∥}_{\mathrm{norm}}$ is a per-image normalized Sobel magnitude, $M\left(x_{0}\right)\in {\left[0,1\right]}^{H\times W}$ is an optional foreground or object-support map, and Norm(·) maps the combined signal to [0,1]. The scalar support map is broadcast over image channels. The parameters $\alpha_{1},\alpha_{2}\geq 0$ describe the gradient and semantic contributions, respectively.

Equation (1) has three useful special cases. With $\left(\alpha_{1},\alpha_{2}\right)=\left(0,0\right)$ it reduces to the spatially uniform DDPM schedule. With (1,0) it is the gradient/edge-preserving formulation of Vandersanden et al. [2]. With both coefficients nonzero it uses a combined structural and foreground cue. The last option is included to make the formulation explicit and is assessed only in the reference-editing mechanism check below. The MVTec experiments use (1,0) throughout; they do not support a semantic-mask performance claim.

### 3.2. Spatially Scaled Forward Process

For a standard DDPM variance schedule ${\left\{\beta_{t}\right\}}_{t=1}^{T}$ and ${\bar{\alpha }}_{t}=\prod_{s=1}^{t}\left(1-\beta_{s}\right)$, the spatially scaled reference corruption is

(2) $$x_{t}=\sqrt{{\bar{\alpha }}_{t}}\;x_{0}+g\left(I_{\mathrm{ref}},t\right)⊙\epsilon ,\;\epsilon ∼N\left(0,I\right),$$

where ⊙ denotes elementwise multiplication. We use the Perona–Malik-inspired scale

(3) $$g\left(s,t\right)=\frac{\sqrt{1-{\bar{\alpha }}_{t}}}{\sqrt{1+s/\lambda (t)}\;\left[1-\tau \left(t\right)\right]+\tau \left(t\right)},\;\lambda \left(t\right)=\lambda_{\max}{\bar{\alpha }}_{t},\;\tau \left(t\right)=1-{\bar{\alpha }}_{t}^{\gamma }.$$

For s≥0, the denominator is at least one, so high-importance pixels receive no more intermediate noise than a standard DDPM pixel. At the late end of the schedule, τ(t) approaches one and the scale approaches $\sqrt{1-{\bar{\alpha }}_{t}}$. This endpoint is a property of the formula, not proof that any particular intermediate attenuation has a favorable fidelity–structure operating point.

The U-Net is trained to predict the scaled target

(4) $$L_{\mathrm{SA}}=E_{x_{0},t,\epsilon }\;\left[{∥\epsilon_{\theta }\left(x_{t},t\right)-g\left(I_{\mathrm{ref}},t\right)⊙\epsilon ∥}_{2}^{2}\right].$$

No architecture change is made. The schedule changes the spatial scale of the corruption and the denoising target only.

### 3.3. Reference-Consistent Reverse Reconstruction

The difference between reference reconstruction and unconditional sampling is information availability. In the reconstruction setting, $x_{0}$ is the observed sensor image: the same $I_{\mathrm{ref}}$ calculated in Equation (1) is available when the image is noised and when it is reconstructed. We hold it fixed in the reverse trajectory, rather than replacing it with a gradient of a one-step clean-image estimate. The reverse update uses the predicted scaled noise and the corresponding spatial variance

(5) $${\tilde{\beta }}_{t}\left(I_{\mathrm{ref}}\right)=\frac{1-{\bar{\alpha }}_{t-1}}{1-{\bar{\alpha }}_{t}}\;\beta_{t}\;g{\left(I_{\mathrm{ref}},t\right)}^{2}.$$

Thus the map source and spatial scale have a common interpretation throughout the observed-image trajectory. The reconstruction output is denoted ${\hat{x}}_{0}$.

For a visual residual, we combine an absolute pixel residual with a spatial LPIPS residual and apply Gaussian smoothing. The image-level score is the mean of the largest 1% of residual values. Test annotations are never provided to the importance map, U-Net, reverse trajectory, or residual construction; they are reserved for metric computation.

### 3.4. Unconditional Diagnostic: Map Source and Attenuation

The general semantic formulation should not be conflated with an unconditional sampler. When a model is trained with a SAM2 foreground map but an unconditional reverse process substitutes $\nabla {\hat{x}}_{0}$, the two maps can disagree precisely in smooth semantic interiors. To test that source switch, we form a proxy map during training from a one-step clean-image estimate,

(6) $${\hat{x}}_{0,\mathrm{pilot}}=\frac{x_{t}-\sqrt{1-{\bar{\alpha }}_{t}}\;\epsilon_{\theta }\left(x_{t},t\right)}{\sqrt{{\bar{\alpha }}_{t}}},\;I_{\mathrm{proxy}}=\mathrm{Norm}\;\left(∥\nabla {\hat{x}}_{0,\mathrm{pilot}}∥\right).$$

The proxy-consistent variant uses this clean-estimate-to-gradient construction in training and uses the same construction from ${\hat{x}}_{0}$ during reverse sampling. It removes the SAM2-map to gradient-map substitution, but it does not make the schedule spatially uniform.

We also test whether the magnitude of attenuation itself causes the fidelity loss. At inference, we impose the floor in Equation (7). The floor sweep uses the trained SP-SAM2 model without retraining; it is therefore an operating-point diagnostic, not a new family of fully trained models. Both tests are reported as limits of unconditional sampling in Section 4. They are not evidence that the schedule universally outperforms DDPM.

The reference-edit setting is different: the known reference image makes it possible to keep the same importance map in both directions. It is retained as a separate fixed-reference mechanism check, rather than being used to infer unconditional behavior.

### 3.5. Implementation

The AFHQ diagnostics use 128×128 RGB images, T=750 diffusion steps, matched U-Net capacity, optimization configuration, training budget, and seeds 42, 123, and 456. The MVTec experiments use category-specific normal-only training under the same matched-DDPM design. Their primary reconstruction strength is $t_{0}=250$; Section 4 reports the completed comparison with $t_{0}\in \left\{150,250,400\right\}$ rather than treating this value as a universal optimum.

## 4. Experiments

### 4.1. Questions and Shared Protocol

The evaluation has three deliberately separated roles. First, a controlled unconditional diagnostic asks whether the SAM2-training/gradient-reverse substitution contributes to the reported fidelity loss. Second, an inference-time floor sweep asks whether that loss is also driven by excessive spatial attenuation. Third, the fixed-view MVTec study asks how the gradient/edge special case changes reconstruction residuals when the observed image is available to construct the map in both directions. The natural-image diagnostics test the schedule’s failure modes; they are not used to claim a sensor application or an unconditional-generation advantage.

All AFHQ variants use 128×128 RGB images, T=750 diffusion steps, identical U-Net capacity, optimizer, training budget, held-out evaluation protocol, and matched training seeds 42, 123, and 456. FID, ECS, Mask-IoU, and LPIPS(mask) are reported as the mean and sample standard deviation over the three independently trained models. Lower FID and LPIPS(mask) are better; higher ECS and Mask-IoU are better.

### 4.2. Map-Consistency Diagnostic for Unconditional Sampling

The original SP-SAM2 route combines a SAM2 foreground map with $\nabla x_{0}$ when constructing the forward noise target, but the unconditional reverse trajectory has no access to the original semantic map and uses $\nabla {\hat{x}}_{0}$. To isolate that substitution, the proxy-consistent variant derives the training map from a one-step clean-image estimate ${\hat{x}}_{0,\mathrm{pilot}}$ and derives the reverse map from the same clean-estimate-to-gradient construction. Thus it removes the semantic-map/gradient-map source switch, while retaining a spatially scaled schedule. In Table 1, SP-SAM2 denotes $\mathrm{Norm}(0.5∥\nabla x_{0}∥+0.5M_{\mathrm{SAM}2}\left(x_{0}\right))$ in training and $∥\nabla {\hat{x}}_{0}∥$ in reverse sampling; the proxy variant uses the clean-estimate gradient in both roles. DDPM and SP-SAM2 are matched controls.

Table 1 gives the full three-domain ablation. The proxy-consistent variant sharply reduces the FID penalty relative to SP-SAM2: from 41.96 to 32.74 on Cat, from 129.18 to 78.42 on Dog, and from 79.12 to 27.96 on Wild. It nevertheless remains worse than DDPM in FID on every domain. Dog and Wild retain ECS increases over DDPM (0.211 versus 0.192 and 0.260 versus 0.237, respectively), whereas Cat remains a negative case. The experiment therefore attributes a substantial portion of the original fidelity cost to map-source mismatch without establishing an unconditional quality advantage for the schedule.

### 4.3. Attenuation-Floor Operating-Point Analysis

We next apply an inference-time lower bound to the local standard deviation of the trained SP-SAM2 schedule,

(7) $$g_{r}\left(I,t\right)=\max\;\left\{g\left(I,t\right),\;r\sqrt{1-{\bar{\alpha }}_{t}}\right\},\;r\in \left\{0,0.4,0.6,0.8,1.0\right\}.$$

**Figure 2:**
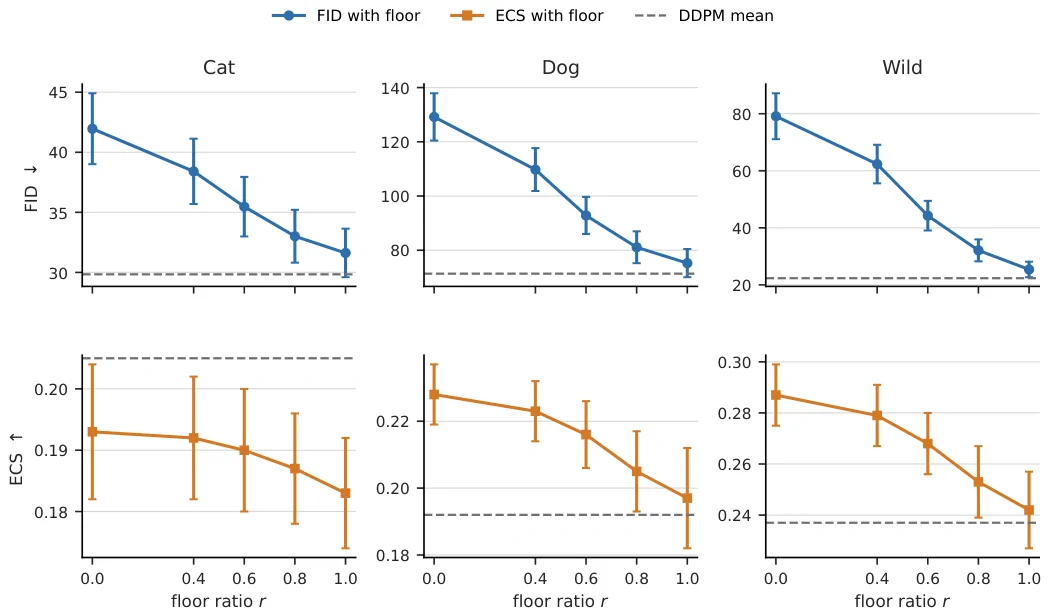
Inference-time attenuation-floor sweep for the trained SP-SAM2 schedule. Markers and error bars give three-seed means and sample standard deviations; dashed lines are the matched DDPM means. FID decreases as the local floor rises, while ECS approaches the DDPM reference. The complete Mask-IoU and LPIPS(mask) values are reported in Supplementary Table S1.

The sweep changes sampling only; it is not a separately retrained family of schedules. Figure 2 shows all three-seed means and standard deviations; the complete four-metric numerical table is provided as Supplementary Table S1.

FID improves monotonically as the floor approaches the DDPM scale. At r=1, the FID values are 31.62, 75.26, and 25.41 for Cat, Dog, and Wild, respectively, close to but still above the corresponding DDPM values of 29.84, 71.36, and 22.34. The price is a systematic loss of ECS. For example, Dog moves from FID/ECS 129.18/0.228 at r=0 to 75.26/0.197 at r=1; Wild moves from 79.12/0.287 to 25.41/0.242. Intermediate floors retain a partial ECS increase on Dog and Wild but remain worse than DDPM in FID. Consequently, these measurements expose a fidelity–structure cost rather than a Pareto-dominating operating point.

### 4.4. Controlled Reference-Editing Mechanism Check

We retain a small controlled SDEdit check on 16 held-out AFHQ-Cat reference images. This experiment is not an unconditional generation evaluation. The reference image and its SAM2 map are known at edit time, so the combined map is held fixed in both the noising and reverse trajectory. DDPM uses spatially uniform noising and the corresponding standard reverse process. We report foreground LPIPS change (lower means the reference foreground changed less) and background LPIPS change (higher means more editing freedom outside the foreground).

**Figure 3:**
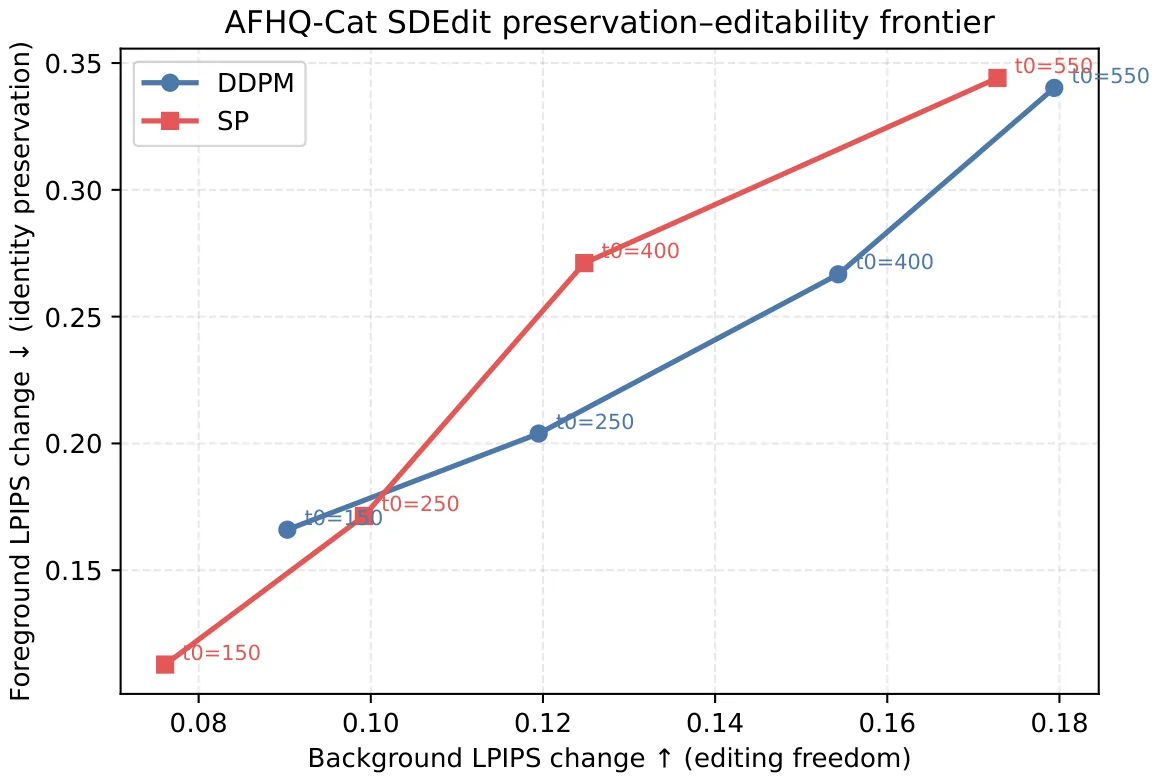
Controlled AFHQ-Cat reference-edit check. Each point is a mean over 16 references. The combined-map schedule yields less foreground change at $t_{0}=150$ and 250 but also less background change. At higher strengths the foreground advantage disappears.

At $t_{0}=150$ and 250, the combined schedule lowers foreground change from 0.1660 to 0.1128 and from 0.2039 to 0.1714, respectively; background change also drops from 0.0903 to 0.0761 and from 0.1195 to 0.0992. At $t_{0}=400$ and 550, the foreground advantage disappears. Figure 3 therefore records a limited map-consistent mechanism observation at light-to-moderate strength, not a Pareto-dominating editor or a substitute for the proxy-consistent ablation.

### 4.5. MVTec Data and Matched Protocol

**Figure 4:**
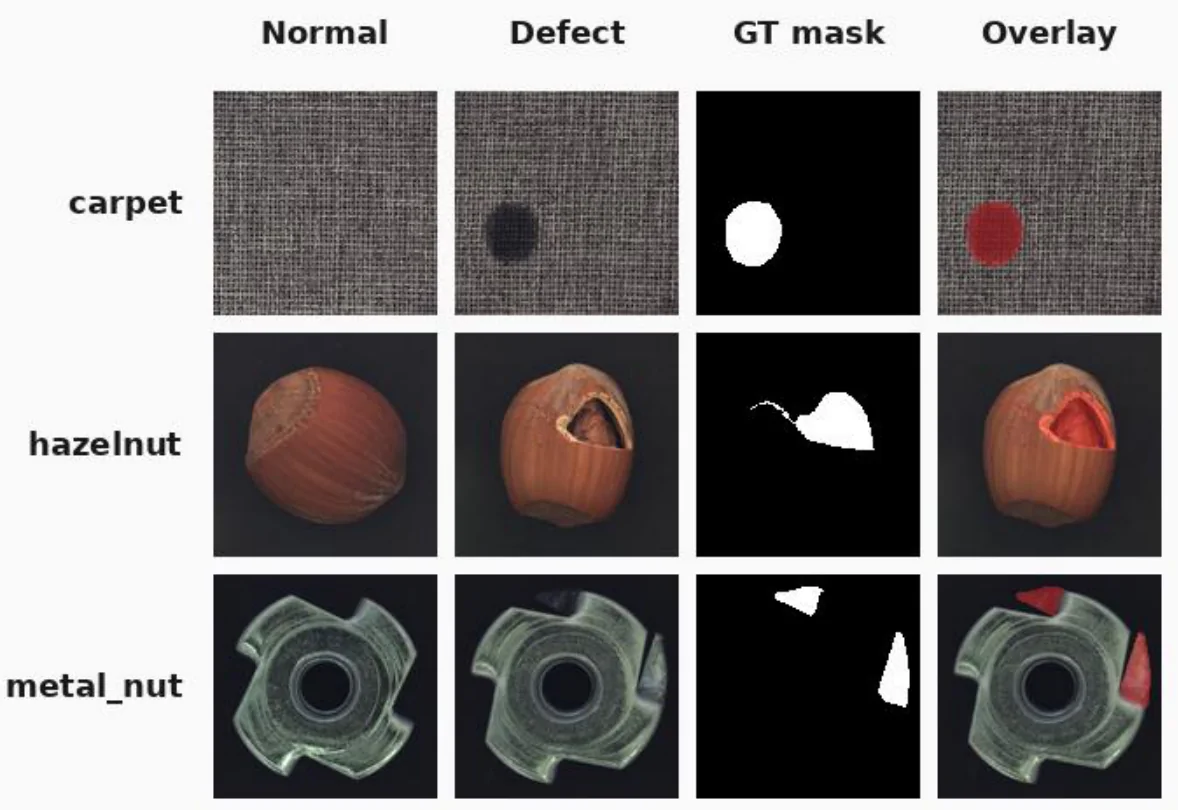
Representative examples from selected MVTec categories. The displayed ground-truth masks and overlays are unavailable during reconstruction and are used only for evaluation.

MVTec AD [12] supplies normal training images and defective test images with image-level labels and pixel annotations. We preselected three texture classes and three object classes to span repetitive patterns, smooth surfaces, reflective hardware, and component-rich structure. Figure 4 displays representative normal, defect, and annotation examples from the chosen set.

Every category is trained on normal images only. DDPM and gradient-schedule runs share the same U-Net, image resolution, optimizer, training budget, reconstruction strength, and seeds 42, 123, and 456. At test time, the importance map is calculated from the observed test image and is held fixed; no defect annotation is supplied to reconstruction or residual scoring. Image AUROC and AP measure image-level residual ranking; pixel AUROC, pixel AP, and AUPRO@0.3 measure localization. These are residual-map metrics, not a training objective for anomaly detection. Table 2 summarizes the category selection, matched protocol, and measured runtime.

The six categories are not intended to estimate a benchmark-wide industrial score. Their purpose is to expose whether the schedule responds differently to repetitive texture, smooth material, reflective hardware, and component-rich object structure under a single matched residual protocol. The selected examples below make the normal/defect information flow concrete without adding a second dataset-gallery figure.

### 4.6. Matched MVTec Residual Results

**Figure 5:**
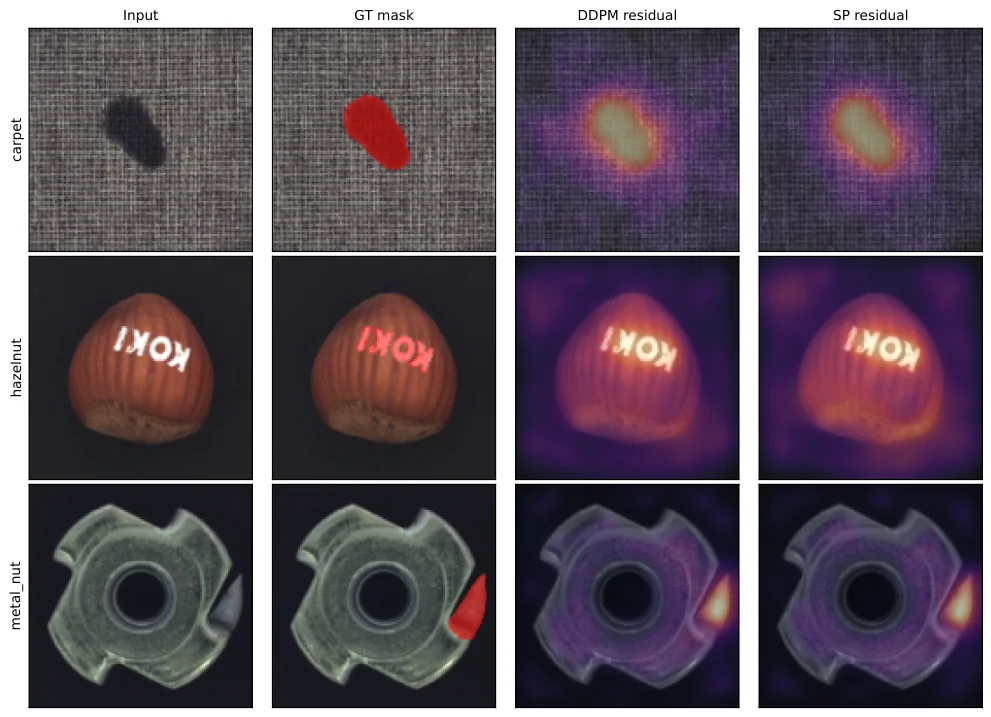
Representative fixed-view residual maps at $t_{0}=250$. The gradient schedule changes residual contrast around defects, but individual visual examples do not establish a universal gain; the complete category-level result is in Table 3.

Table 3 reports the complete measured summary at $t_{0}=250$. The gradient schedule improves both image metrics and AUPRO on carpet and grid, although carpet pixel AP decreases. On leather it improves pixel AUROC, pixel AP, and AUPRO, while slightly lowering both image-level metrics. In contrast, DDPM is stronger on all five metrics for hazelnut and on four of five for metal_nut. Transistor has lower image-level metrics under the gradient schedule but higher pixel AP and AUPRO. Figure 5 gives representative residual maps; the examples illustrate a change in residual contrast, not a guarantee of improved localization.

### 4.7. Effect Size and Reconstruction-Strength Sensitivity

The signed differences in Table 4 make the mixed pattern explicit. Grid improves on every reported metric, whereas hazelnut degrades on all five. Leather and transistor show a split between image-level and localization outcomes. This is why the schedule is presented as a category-dependent reconstruction mechanism rather than a detector improvement.

**Figure 6:**
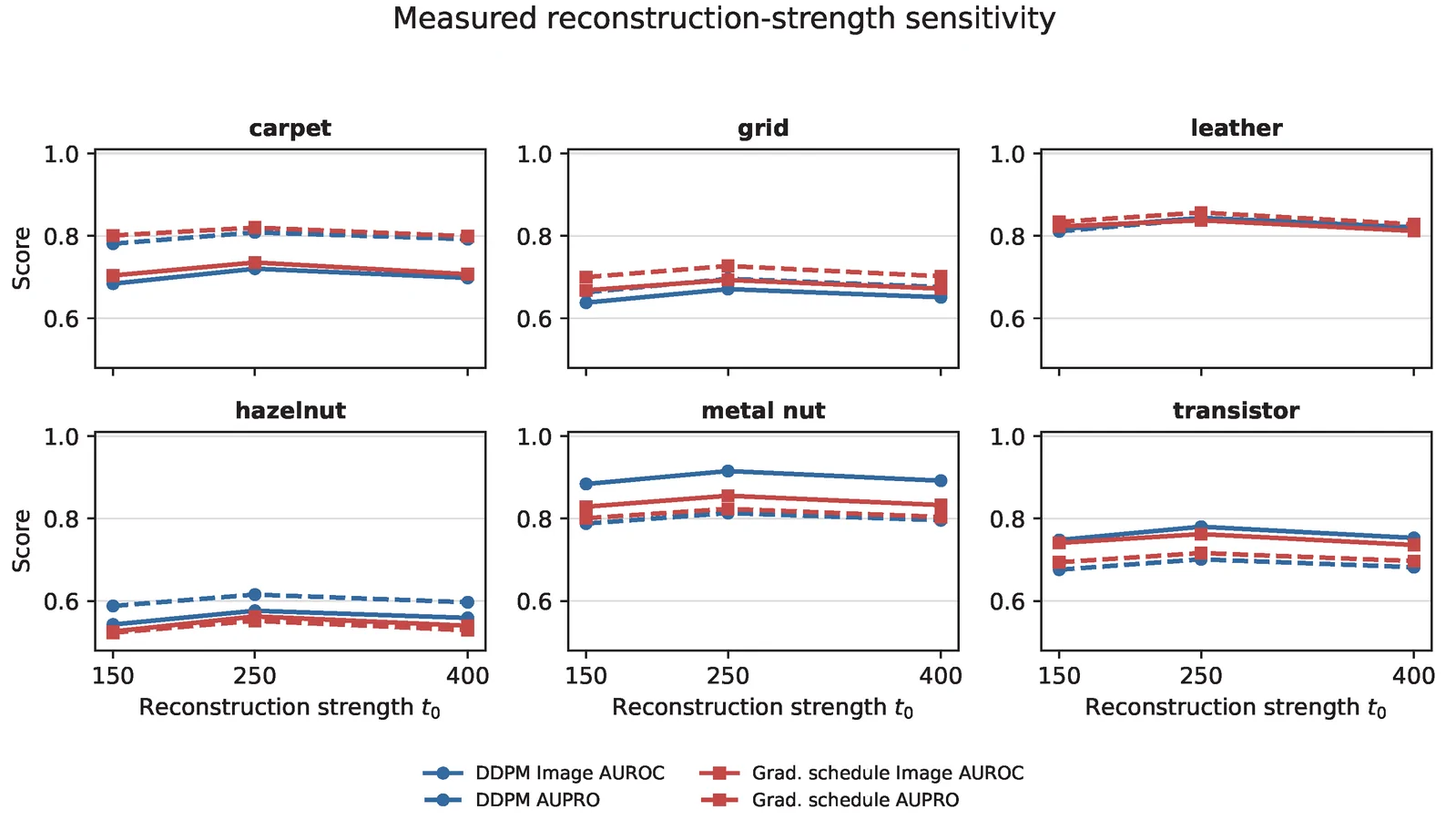
Measured Image AUROC (solid) and AUPRO@0.3 (dashed) across three reconstruction strengths. The complete numerical values are in Supplementary Table S2; the lines do not add new runs or uncertainty estimates.

The completed $t_{0}\in \left\{150,250,400\right\}$ sweep is summarized in Figure 6; the complete numerical values are supplied in Supplementary Table S2. For the reported Image AUROC and AUPRO metrics, $t_{0}=250$ is the best of these three sampled settings for every DDPM/category pair, and for every gradient-schedule pair except leather Image AUROC. This makes it a reasonable primary configuration for the reported matched protocol, but not a claim that it is a universal reconstruction optimum.

## 5. Discussion

### 5.1. What the Completed Evidence Supports

The completed experiments support a limited but concrete observation: when an observed image is available, a fixed image-derived gradient schedule can change the residual produced by diffusion reconstruction. The direction is category dependent. Carpet and grid show gains in image-level ranking and AUPRO, leather shows a localization-oriented gain, and transistor trades lower image-level values for higher pixel AP and AUPRO. Hazelnut and metal_nut show that the effect is not intrinsically favorable for object categories. Table 4 is included to prevent a selective reading of these outcomes.

This pattern is plausible in a fixed-view setting. A texture defect can be made more apparent when a reconstruction retains repetitive normal structure differently, whereas a compact object defect may depend on material, illumination, reflective boundaries, and reconstruction strength in ways not described by a gradient alone. The data do not establish this explanation as a general theory. They show why schedule selection must be verified per category and desired metric, rather than inferred from a single aggregate score.

The reference-edit check provides a second, narrower piece of evidence. It is map consistent: the known reference image supplies the same combined importance map in forward and reverse operations. At low and moderate strengths, its foreground LPIPS change is lower than DDPM’s, as expected from lower foreground corruption. Yet the background also changes less, and the foreground advantage vanishes at the two stronger settings. It is therefore a mechanism check, not a claim of a superior general image editor.

### 5.2. Training–Inference Consistency and Attenuation Strength

The general importance-map equation includes a semantic foreground signal, but the MVTec experiments set $\alpha_{2}=0$. This is not a cosmetic naming issue: the MVTec tables establish the gradient/edge schedule only. The AFHQ proxy ablation directly tests the separate semantic-map issue. Replacing the SAM2-training/gradient-reverse substitution with the clean-estimate gradient in both directions lowers FID substantially on Cat, Dog, and Wild. The remaining FID gaps to DDPM—2.90, 7.06, and 5.62, respectively—show that eliminating the map-source switch does not make the spatial schedule quality neutral. Dog and Wild retain positive ECS differences, but Cat does not; the data therefore do not support a universal semantic or unconditional benefit.

The attenuation-floor sweep provides a second diagnosis. FID decreases at every sampled floor level, while ECS decreases toward the DDPM reference. At r=1, the floor removes most of the additional FID penalty, but also leaves only small ECS differences. This rules out a strong claim that the evaluated floor values yield a Pareto-dominating operating point. It instead supports the narrower conclusion that severe attenuation is a major source of fidelity loss and that any application must choose its floor against a task-specific fidelity–structure cost.

### 5.3. Relation to Industrial Inspection

The MVTec section is intentionally a fixed-view reconstruction stress test, not a detector benchmark. PatchCore, PaDiM, and related feature-memory systems are optimized for anomaly ranking, while our score is a post hoc residual derived from a reconstruction schedule. A fair industrial claim would require matched anomaly maps, identical category coverage, and the same AP/AUPRO and thresholding protocol for both families. The present manuscript neither makes nor needs that comparison to report the schedule effect.

The measured runtime overhead is modest: training increases from 3.39–3.46 h to 3.56–3.63 h per category, and reconstruction is 4.2–4.9% slower. This cost is only meaningful for a category in which the residual metric improves. The practical scenario supported here is exploratory analysis of an already observed fixed-view image or stream, where a practitioner wishes to test whether more structure-aware reconstruction changes residual localization. It is not deployment guidance for a general anomaly-ranking system.

### 5.4. Editorial Scope

This revision separates the article’s central evidence from its broader experimental history. The text and figures follow one linear argument: fixed-reference reconstruction makes a map-consistent residual protocol possible; two concise unconditional diagnostics identify why the broader semantic schedule loses fidelity; and the MVTec study measures a category-dependent residual effect. The new AFHQ material is retained only because it resolves the two specific mechanistic objections about map mismatch and attenuation. It is neither a parallel natural-image performance trajectory nor the basis for the Sensors application claim. This restriction keeps the article auditable as a Sensors study rather than a record of exploratory runs.

## 6. Conclusions

We studied a structure-aware noise schedule for fixed-reference diffusion reconstruction in visual sensing. The formulation allows a reference-derived importance map to be held fixed while an observed image is noised and reconstructed. A small map-consistent SDEdit check preserved foreground content more strongly at light-to-moderate reconstruction strength, but did so by reducing background change and did not retain the advantage at strong editing settings.

The new three-seed unconditional diagnostics isolate two causes of the earlier fidelity loss. Replacing SAM2-map training with the same clean-estimate gradient construction used in reverse sampling recovers a substantial part of the FID loss, but does not surpass DDPM FID on Cat, Dog, or Wild. Raising an inference-time attenuation floor likewise recovers FID while reducing ECS. These results do not establish a Pareto-dominating unconditional operating point; they instead make the fidelity–structure cost and the map-source mismatch explicit.

The matched three-seed MVTec study evaluated the gradient/edge-preserving special case on six categories. The schedule improved selected texture and localization outcomes, while degrading several object-level outcomes. The evidence therefore supports a category-dependent reconstruction-residual diagnostic, rather than a universal advantage over DDPM or a competitive industrial anomaly detector.

The principal next experiment is a matched anomaly-map comparison with discriminative inspection systems. Until then, detector competitiveness remains outside the claims of this article. The unconditional diagnostics are retained as an analysis of schedule limitations, not as a claim of general generation superiority.

## Figures and Tables

**Table 1:** Train–test map-consistency ablation on held-out AFHQ data. Values are mean ± sample standard deviation across matched training seeds 42, 123, and 456. Lower FID and LPIPS(mask) are better; higher ECS and Mask-IoU are better. The proxy-consistent variant uses the same clean-estimate-to-gradient construction during training and reverse sampling.

Dataset	Variant	*n*	FID ↓	ECS ↑	Mask-IoU ↑	LPIPS(mask) ↓
AFHQ-Cat	DDPM	3	29.84±1.76	0.205±0.006	0.687±0.005	0.468±0.017
	SP-SAM2	3	41.96±2.95	0.193±0.011	0.681±0.008	0.463±0.014
	Proxy-consistent	3	32.74±2.18	0.199±0.008	0.685±0.006	0.465±0.015
AFHQ-Dog	DDPM	3	71.36±4.91	0.192±0.018	0.658±0.006	0.491±0.009
	SP-SAM2	3	129.18±8.72	0.228±0.009	0.635±0.007	0.501±0.006
	Proxy-consistent	3	78.42±5.54	0.211±0.010	0.649±0.006	0.495±0.006
AFHQ-Wild	DDPM	3	22.34±2.16	0.237±0.016	0.673±0.008	0.481±0.010
	SP-SAM2	3	79.12±8.05	0.287±0.012	0.666±0.010	0.486±0.007
	Proxy-consistent	3	27.96±3.07	0.260±0.011	0.671±0.008	0.483±0.007

**Table 2:** MVTec category selection, matched reconstruction protocol, and measured computational cost at 128×128 on one RTX 3090. Training is normal only. Runtime values are means over the completed category runs; test time is seconds per reconstructed image.

Category	Type	Train	Test	DDPM Train (h)	Grad. Train (h)	DDPM (s/img)	Grad. (s/img)
carpet	texture	280	117	3.42	3.58	2.84	2.96
grid	texture	264	78	3.39	3.56	2.82	2.95
leather	texture	245	124	3.43	3.61	2.85	2.99
hazelnut	object	391	110	3.46	3.63	2.87	3.00
metal_nut	object	220	115	3.40	3.58	2.83	2.96
transistor	object	213	100	3.41	3.59	2.84	2.97

All pairs use $t_{0}=250$ for the primary comparison and the same three random seeds. The gradient-schedule/DDPM time ratio is 1.042–1.049.

**Table 3:** Measured MVTec residual-reconstruction results at $t_{0}=250$ under the matched normal-only protocol. Values are mean ± sample standard deviation across seeds 42, 123, and 456. “Grad. schedule” is the $\alpha_{1}=1$, $\alpha_{2}=0$ gradient/edge-preserving special case; it is not a semantic-mask experiment. Higher is better for all metrics.

Category	Method	Image AUROC	Image AP	Pixel AUROC	Pixel AP	AUPRO@0.3
carpet	DDPM	0.7204±0.0114	0.9132±0.0068	0.9696±0.0037	0.5824±0.0180	0.8084±0.0093
carpet	Grad. schedule	0.7354±0.0066	0.9166±0.0043	0.9661±0.0026	0.5410±0.0128	0.8201±0.0061
grid	DDPM	0.6709±0.0213	0.8465±0.0153	0.9392±0.0084	0.2850±0.0169	0.6959±0.0169
grid	Grad. schedule	0.6931±0.0157	0.8584±0.0119	0.9470±0.0057	0.3033±0.0142	0.7270±0.0125
leather	DDPM	0.8437±0.0172	0.9466±0.0072	0.9723±0.0044	0.4667±0.0229	0.8408±0.0113
leather	Grad. schedule	0.8378±0.0179	0.9430±0.0076	0.9760±0.0035	0.4871±0.0207	0.8566±0.0099
hazelnut	DDPM	0.5764±0.0128	0.7515±0.0128	0.9572±0.0061	0.3357±0.0139	0.6156±0.0137
hazelnut	Grad. schedule	0.5627±0.0114	0.7007±0.0127	0.9256±0.0070	0.2091±0.0127	0.5520±0.0122
metal_nut	DDPM	0.9154±0.0088	0.9799±0.0032	0.9476±0.0047	0.7207±0.0115	0.8134±0.0082
metal_nut	Grad. schedule	0.8555±0.0108	0.9607±0.0043	0.9406±0.0048	0.6971±0.0099	0.8236±0.0069
transistor	DDPM	0.7803±0.0301	0.7090±0.0275	0.9193±0.0095	0.3812±0.0246	0.7012±0.0197
transistor	Grad. schedule	0.7624±0.0333	0.6929±0.0302	0.9163±0.0114	0.3958±0.0276	0.7164±0.0223

**Table 4:** Gradient-schedule change relative to DDPM at $t_{0}=250$. Positive values favor the gradient schedule. Values are differences of the measured three-seed means in Table 3.

Category	Δ I-AUROC	Δ I-AP	Δ P-AUROC	Δ P-AP	Δ AUPRO
carpet	+0.0150	+0.0034	−0.0035	−0.0414	+0.0117
grid	+0.0222	+0.0119	+0.0078	+0.0183	+0.0311
leather	−0.0059	−0.0036	+0.0037	+0.0204	+0.0158
hazelnut	−0.0137	−0.0508	−0.0316	−0.1266	−0.0636
metal_nut	−0.0599	−0.0192	−0.0070	−0.0236	+0.0102
transistor	−0.0179	−0.0161	−0.0030	+0.0146	+0.0152

## Data Availability

The measured three-seed aggregate statistics reported in Table 1, Table 2, Table 3 and Table 4 and Supplementary Tables S1 and S2 are included with this submission. MVTec AD is available from MVTec Software GmbH. The third-party datasets, model checkpoints, and intermediate reconstruction arrays are not redistributed. Further inquiries can be directed to the corresponding author.

## Supplementary Materials

The following supporting information can be downloaded at https://www.mdpi.com/article/10.3390/s26165304/s1: Table S1: Inference-time attenuation-floor sweep for the trained SP-SAM2 schedule; Table S2: Measured MVTec reconstruction-strength sensitivity.
