# Supplementary material for: Structure-Aware Noise Scheduling for Fixed-View Visual Sensor Reconstruction
Source: Sensors (Basel). 2026 Aug 21;26(16):5304. doi: 10.3390/s26165304 (PMC13517880; doi:10.3390/s26165304)
Supplement: Supplementary file 1 [file sensors-26-05304-s001.zip › sensors-4477624-supplementary.pdf]

Supplementary Tables S1–S2

Structure-Aware Noise Scheduling for Fixed-View Visual Sensor Reconstruction

The floor is $g_r(I,\ t) = \max\{g(I,\ t),\ r\sqrt{1-\bar{\alpha}_t}\}$. Values are mean ± sample standard deviation across matched training seeds 42, 123, and 456. This is a sampler operating-point analysis, not a separately retrained family of schedules.

Table S1. Inference-time attenuation-floor sweep for the trained SP-SAM2 schedule.

Dataset	r	n	FID ↓	ECS ↑	Mask-IoU ↑	LPIPS(mask) ↓
AFHQ-Cat	0.0	3	41.96 ± 2.95	0.193 ± 0.011	0.681 ± 0.008	0.463 ± 0.014
	0.4	3	38.41 ± 2.72	0.192 ± 0.010	0.682 ± 0.008	0.464 ± 0.014
	0.6	3	35.47 ± 2.48	0.190 ± 0.010	0.683 ± 0.007	0.465 ± 0.015
	0.8	3	33.01 ± 2.20	0.187 ± 0.009	0.685 ± 0.006	0.467 ± 0.016
	1.0	3	31.62 ± 2.02	0.183 ± 0.009	0.686 ± 0.006	0.468 ± 0.016
AFHQ-Dog	0.0	3	129.18 ± 8.72	0.228 ± 0.009	0.635 ± 0.007	0.501 ± 0.006
	0.4	3	109.76 ± 7.91	0.223 ± 0.009	0.639 ± 0.007	0.499 ± 0.006
	0.6	3	92.84 ± 6.83	0.216 ± 0.010	0.645 ± 0.006	0.496 ± 0.006
	0.8	3	81.10 ± 5.88	0.205 ± 0.012	0.651 ± 0.006	0.493 ± 0.007
	1.0	3	75.26 ± 5.20	0.197 ± 0.015	0.656 ± 0.006	0.492 ± 0.008
AFHQ-Wild	0.0	3	79.12 ± 8.05	0.287 ± 0.012	0.666 ± 0.010	0.486 ± 0.007
	0.4	3	62.35 ± 6.76	0.279 ± 0.012	0.667 ± 0.010	0.485 ± 0.007
	0.6	3	44.26 ± 5.19	0.268 ± 0.012	0.669 ± 0.009	0.484 ± 0.007
	0.8	3	32.09 ± 3.85	0.253 ± 0.014	0.671 ± 0.008	0.482 ± 0.008
	1.0	3	25.41 ± 2.72	0.242 ± 0.015	0.672 ± 0.008	0.481 ± 0.009

Values are three-seed means. Higher is better.

Table S2. Measured MVTec reconstruction-strength sensitivity.

Category	Method	Image AUROC			AUPRO@0.3		
		t_0 = 150	t_0 = 250	t_0 = 400	t_0 = 150	t_0 = 250	t_0 = 400
carpet	DDPM	0.6840	0.7204	0.6980	0.7810	0.8084	0.7920
carpet	Grad. schedule	0.7040	0.7354	0.7070	0.8010	0.8201	0.7990
grid	DDPM	0.6380	0.6709	0.6510	0.6630	0.6959	0.6760
grid	Grad. schedule	0.6680	0.6931	0.6720	0.7000	0.7270	0.7020
leather	DDPM	0.8160	0.8437	0.8210	0.8110	0.8408	0.8190
leather	Grad. schedule	0.8230	0.8378	0.8120	0.8340	0.8566	0.8280
hazelnut	DDPM	0.5430	0.5764	0.5590	0.5880	0.6156	0.5970
hazelnut	Grad. schedule	0.5260	0.5627	0.5400	0.5230	0.5520	0.5290
metal_nut	DDPM	0.8840	0.9154	0.8920	0.7880	0.8134	0.7960
metal_nut	Grad. schedule	0.8290	0.8555	0.8330	0.8010	0.8236	0.8040
transistor	DDPM	0.7480	0.7803	0.7530	0.6760	0.7012	0.6820
transistor	Grad. schedule	0.7410	0.7624	0.7360	0.6940	0.7164	0.6970
